# Draft Genome Sequences of 37 *Salmonella enterica* Strains Isolated from Poultry Sources in Nigeria

**DOI:** 10.1128/genomeA.00315-16

**Published:** 2016-05-05

**Authors:** Nicodemus M. Useh, Emmanuel O. Ngbede, Nguavese Akange, Milton Thomas, Andrew Foley, Mitchel Chan Keena, Eric Nelson, Jane Christopher-Hennings, Masaru Tomita, Haruo Suzuki, Joy Scaria

**Affiliations:** a Department of Veterinary Pathology and Microbiology, University of Agriculture, Makurdi, Nigeria; b Department of Veterinary and Biomedical Sciences, South Dakota State University, Brookings, South Dakota, USA; c Institute for Advanced Biosciences, Keio University, Fujisawa, Kanagawa, Japan

## Abstract

Here, we report the availability of draft genomes of several *Salmonella* serotypes, isolated from poultry sources from Nigeria. These genomes will help to further understand the biological diversity of *S. enterica* and will serve as references in microbial trace-back studies to improve food safety.

## GENOME ANNOUNCEMENT

Nontyphoidal salmonellosis is one of the common causes of bacterial diarrhea worldwide (1). Globally, 94 million cases of gastroenteritis and 115,000 deaths each year are estimated to be caused by nontyphoidal salmonellosis (2). While North America and Europe have constituted active salmonella surveillance programs, very limited epidemiological data are available in developing countries, particularly in sub-Saharan Africa. Therefore, we have conducted an epidemiological investigation of *Salmonella enterica* prevalence in poultry samples from South-West and North-Central Nigeria in 2014. Here, we report the draft genome sequences of the 37 *S. enterica* strains, isolated as part of this study (Table 1).

**TABLE 1:** Characteristics of 37 *Salmonella enterica* strains, isolated from poultry sources in Nigeria

Strain name	Accession no.	Serotype	Genome size (bp)	G+C content (%)	No. of proteins
NGUA01	BCNZ00000000	Kentucky	4,881,836	52.2	4,524
NGUA02	BCOA00000000	Kentucky	4,876,582	52.2	4,534
NGUA03	BCOB00000000	Herston	4,887,324	52.1	4,567
NGUA04	BCOC00000000	Herston	4,853,780	52.2	4,508
NGUA05	BCOD00000000	13:d:e,n,z15	4,707,314	52.2	4,350
NGUA06	BCOE00000000	Zega	4,680,950	52.2	4,294
NGUA07	BCOF00000000	Kentucky	4,950,201	52.1	4,571
NGUA08	BCOG00000000	Zega	4,723,274	52.2	4,325
NGUA09	BCOH00000000	Herston	4,828,175	52.2	4,495
NGUA10	BCOI00000000	Herston	5,059,871	52	4,692
NGUA12	BCOK00000000	Zega	4,689,083	52.2	4,290
NGUA13	BCOL00000000	Colindale	4,872,659	52.1	4,541
NGUA14	BCOM00000000	Nima	4,536,192	52.3	4,198
NGUA16	BCOO00000000	Kentucky	4,970,350	52.1	4,635
NGUA17	BCOP00000000	Zega	4,687,717	52.2	4,300
NGUA18	BCOQ00000000	Kentucky	5,259,276	52	4,879
NGUA19	BCOR00000000	Zega	4,721,675	52.2	4,350
NGUA20	BCOS00000000	Kentucky	4,871,939	52.2	4,512
NGUA21	BCOT00000000	Kentucky	4,947,233	52.1	4,617
NGUA22	BCOU00000000	Herston	4,801,625	52.1	4,485
NGUA23	BCOV00000000	Kentucky	4,975,494	52.2	4,642
NGUA24	BCOW00000000	Herston	4,847,498	52.1	4,536
NGUA25	BCOX00000000	Zega	4,702,417	52.2	4,310
NGUA26	BCOY00000000	Lingwala	4,783,830	52.1	4,374
NGUA27	BCOZ00000000	Zega	4,875,130	52.2	4,488
NGUA28	BCPA00000000	Kentucky	4,885,381	52.2	4,538
NGUA29	BCPB00000000	Zega	4,699,130	52.2	4,321
NGUA30	BCPC00000000	Kentucky	4,867,532	52.2	4,528
NGUA31	BCPD00000000	Zega	4,731,115	52.2	4,355
NGUA32	BCPE00000000	Kentucky	4,827,432	52.2	4,472
NGUA33	BCPF00000000	13:d:e,n,z15	4,708,185	52.2	4,356
NGUA34	BCPG00000000	Zega	4,682,708	52.2	4,288
NGUA35	BCPH00000000	Herston	4,879,810	52.1	4,557
NGUA36	BCPI00000000	Nima	4,523,766	52.3	4,183
NGUA37	BCPJ00000000	Kentucky	4,873,902	52.2	4,524
NGUA38	BCPK00000000	Livingstone	4,860,227	51.9	4,462
NGUA39	BCPL00000000	Kentucky	4,878,399	52.2	4,505

*S. enterica* strains were isolated by enrichment culture in tetrathionate broth followed by growth on XLT4 agar. The identities of the strains were further confirmed by Matrix-assisted laser desorption ionization time-of-flight (MALDI-TOF). Genomic DNA from each strain was isolated from 1.0 ml of grown cultures using the E.Z.N.A. bacterial DNA kit (Omega Bio-tek, Norcross, GA). Following the manufacturer’s protocol, sequencing libraries were prepared using 1.0 ng of genomic DNA using the Nextera XT kit (Illumina, San Diego, CA). Genomes were sequenced on an Illumina MiSeq platform using V2 paired-end chemistry (2 × 250-bp). *De novo* genome assembly was performed using SPAdes version 3.5.0 (3). Genome annotation was performed using Prokka version 1.11 (4). Genome size and G+C content were estimated with all contigs of each strain. Among the 37 strains, median values for genome size and G+C content were 4.87 (Mb) and 52.2 (%), respectively (Table 1), and were similar to those of the *S. enterica* complete genomes. The serotypes of the isolates were determined using SeqSero (5).

As shown in Table 1, we obtained a diverse collection of *S. enterica* isolates from poultry sources in Nigeria. *S. enterica* serotype Enteritidis*,* the most common serotype associated with poultry worldwide, was not found in our study. The most common serotype among our isolates was *S*. Kentucky, followed by *S.* Zega and *S.* Herston. Other serotypes found were *S.* Nima, *S.* Livingston, and *S*. Colindale. The genomes of several *Salmonella* serotypes that we report here are the first reports, or are relatively rare. For example, only one genome of an *S.* Livingston isolate, from a lake in Canada, has been reported (6). To our knowledge, the genomes described in Table 1 belonging to *S.* Zega, *S.* Herston, *S.* Nima, and *S*. Colindale are the first genomes being reported for these serotypes. *S.* Nima has been reported to have caused an international outbreak through contaminated chocolate (7). *S.* Zega was previously isolated from Zaire (8). *S*. Colindale is one of the common *Salmonella* serotypes isolated from fresh lettuce in Burkina Faso (9). *S.* Herston has been isolated from diarrheal cases in children from Niger. The genomes we report here will help to understand more fully the biological diversity of *S. enterica* and will serve as references in microbial trace-back studies to improve food safety.

### Nucleotide sequence accession numbers.

The sequences have been deposited in DDBJ/EMBL/GenBank under the accession numbers listed in Table 1.
